# Cryptanalysis of an Image Encryption Algorithm Using DNA Coding and Chaos

**DOI:** 10.3390/e27010040

**Published:** 2025-01-07

**Authors:** Yuzhuo Zhao, Qiqin Shi, Qun Ding

**Affiliations:** Electrical Engineering College, Heilongjiang University, Harbin 150080, China; yuzhuoz@163.com (Y.Z.); shiqiqin85@163.com (Q.S.)

**Keywords:** cryptanalysis, chaotic, image encryption, plaintext correlation

## Abstract

In recent years, many chaotic image encryption algorithms have been cracked by chosen plaintext attack. Therefore, the method of associating the key with the plaintext to resist the cryptanalysis has received extensive attention from designers. This paper proposes a new method of cryptanalysis for image encryption algorithms with a key associated with plaintext. We broke an image encryption scheme using chaos and DNA encoding. Through our comprehensive security analysis, we found a security vulnerability in the mechanism of the association between the key and plaintext and proposed a breaking scheme. The experimental results show that the chosen plaintext attack can recover the cipher image to the plain image. The cryptanalysis scheme proposed in this paper can provide new ideas for subsequent cryptanalysis work and also provide some meaningful references for designers to improve the security of encryption algorithms when designing them. In addition, we also propose an improved logistic chaotic map with random bit-position scrambling. The improved chaotic map has a wider parameter range and a larger Lyapunov exponent. In the end, some suggestions are given to improve the original algorithm to resist such attacks.

## 1. Introduction

Since 1998, the American scholar J. Fridrich has been at the vanguard of applying chaos theory to the field of image encryption [1]. The potential of chaos in image encryption has been well demonstrated, and algorithms based on scrambling and diffusion structures have developed rapidly [2,3]. However, in comparison with the established text encryption standards, the emergence of chaotic image encryption has been delayed, and the security analysis of some algorithms is insufficient. Consequently, the security of these algorithms is questionable. The purpose of cryptanalysis is to identify security vulnerabilities in encryption algorithms, obtain the key or equivalent key through various attack schemes, recover part or even all of the plaintext from the corresponding ciphertext, and improve it to enhance the security performance [4,5].

Some image encryption algorithms prove that they are secure by using numeric statistics. Recent studies have demonstrated that while numerical statistical analysis plays an important role in evaluating the security of an image encryption algorithm, it is not a comprehensive indicator of the algorithm’s overall security [6,7]. Essaid et al. devised a novel Hill cipher and developed a chaotic image encryption method based on the Hill cipher and three chaotic maps to enhance security. The presence of equivalent keys within the algorithm is ascertained through meticulous cryptanalysis [8]. Furthermore, it combines chosen plaintext attack (CPA) and chosen ciphertext attack (CCA) [9].

In recent years, there has been a growing interest among researchers in the field of cryptography in the combination of new techniques with chaotic systems as a means to enhance the security of algorithms [10]. However, it should be noted that some encryption algorithms that introduce new mechanisms lack the ability to enhance the security of the algorithm itself [11,12]. In 2024, Wen and Lin demonstrated the feasibility of cracking an image encryption algorithm that combines quantum chaotic and DNA coding techniques [13]. Due to the origin of the encryption algorithm, the existence of equivalent keys, and the absence of confusion and diffusion in the DNA domain, an encryption algorithm can be broken using only four special plain images.

Concurrently, some cryptographic designers propose the use of a mechanism of associating the key with the plaintext to resist CPA [14]. Many image encryption algorithms in which keys are associated with plaintext have also been broken [15,16,17,18]. Patro et al. introduced a multi-image encryption framework that utilizes cross-coupled chaotic mapping [19]. This algorithm uses the hash value after mixing multi-plain images as the initial value of cross-coupled chaos and carries out feedforward scrambling and diffusion operations on the plain images based on the chaotic sequence line by line and column by column. Laiphrakpam et al. [20] proposed a ciphertext-only attack method in 2024, which uses the feedforward XOR operation and the high correlation between rows and columns of permutation images to decipher and recover the plain images without using the key.

According to previous research, we found that many image encryption algorithms utilize the hash value derived from the plain image as the key, and few cryptanalyses have been used to crack this mechanism. This paper presents a comprehensive analysis of the image encryption algorithm proposed by Wang et al. [21], which employs a chaotic map and DNA encoding, named NEIEA-BCD. The algorithm associates the initial key with the hash value of the original image; however, the correlation is found to be weak in this paper. The original algorithm employs DNA operations, which increases the algorithm’s overall complexity. However, the underlying process is fundamentally a two-bit diffusion-only process. In light of the aforementioned security vulnerabilities, this paper employs the CPA method to recover the original plain image by constructing distinct special plain images. Moreover, an enhanced logistic chaotic map [22] is put forth, exhibiting a broader parameter range and a larger Lyapunov exponent (LE) than the logistic chaotic system. Ultimately, an enhanced image encryption algorithm is devised based on the initial encryption algorithm. This paper offers novel insights into the cryptanalysis of image encryption algorithms in which the hash value of the plain image is linked to the key.

This paper is structured as follows: Section 2 provides a description of the original encryption algorithm and points out its security vulnerabilities. Section 3 outlines the cryptanalysis process and presents the experimental results. Section 4 presents enhancements to the encryption algorithm, while Section 5 provides a conclusion to this study.

## 2. Description of NEIEA-BCD

### 2.1. Chaotic Mapping of NEIEA-BCD

NEIEA-BCD employs piecewise linear chaotic mapping (PWLCM) and logistic mapping for the generation of the parameters required for its operation. PWLCM is employed for the construction of the key image, while logistic regression is applied for the selection of specific DNA operations and rules. PWLCM is delineated in Equation (1).

(1) $$x_{i+1}=F_{p}\left(x_{i}\right)=\left\{\begin{array}{c} x_{i}/p,\\ \left(x_{i}-p\right)/\left(0.5-p\right),\\ F_{p}\left(1-x_{i}\right), \end{array}\right.\begin{array}{c} 0<x_{i}<p\\ p\leq x_{i}<0.5\\ 0.5\leq x_{i}<1 \end{array}$$

where x∈(0,1), p∈(0,0.5).

The logistic chaotic map is described by Equation (2).

(2) $$x_{i+1}=\mu x_{i}\left(1-x_{i}\right)$$

where x∈(0,1) and μ∈(3.9,4].

### 2.2. DNA Sequence

Deoxyribonucleic acid (DNA) is composed of four nucleotide bases: A, T, C, and G. In this structure, adenine pairs with thymine, while guanine pairs with cytosine, reflecting their complementary nature. According to the principles of DNA complementarity, there are eight permissible combinations, as illustrated in Table 1.

DNA operations such as Exclusive OR (XOR), addition, and subtraction can be performed between DNA sequences. The specific operational procedures are outlined in Table 2.

### 2.3. MD5 and $x_{0}$

The Message Digest Algorithm 5 (MD5) is capable of mapping any length of data to a 128-bit integer, a process that is irreversible. The MD5 value ($P_{hash}$) of the plain image (*P*) is 128 bits in total. The initial value ($x_{0}$) of the chaotic system is calculated according to Equation (3).

(3) $$x_{0}=\;\mathrm{mod}\;\left(d_{1}⊕d_{2}⊕d_{3}⊕d_{4},256\right)/255\;$$

In NEIEA-BCD, $d_{1}$ represents the first eight bits of $P_{hash}$, $d_{2}$ denotes bits 9–16, $d_{3}$ signifies bits 17–24 of $P_{hash}$, and $d_{4}$ corresponds to bits 25–32 of $P_{hash}$. The initial value, designated as $x_{0}$, is obtained by taking the result of the XORof $d_{1}$, $d_{2}$, $d_{3}$, and $d_{4}$, expressed in modulo 256, and dividing by 255. $x_{0}$ must not be equal to 0 or 1.

### 2.4. Encryption Process of NEIEA-BCD

**Figure 1:**
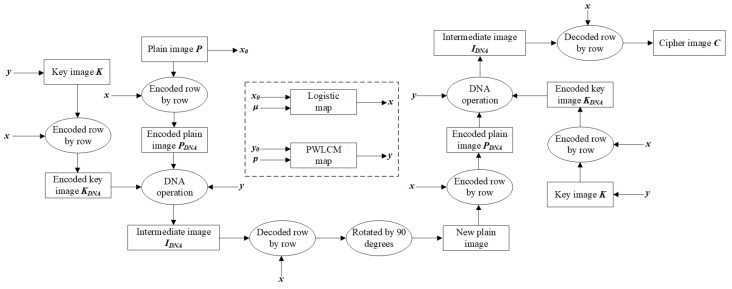
Encryption process.

The following is a detailed account of the specific process of NEIEA-BCD, which encrypts a grayscale image with a resolution of M × N. The overall process is illustrated in Figure 1.

The initial keys of NEIEA-BCD are constituted by the μ parameter, the initial value ($x_{0}$) of the logistic chaotic system, the *p* parameter, and the initial value ($y_{0}$) of the PWLCM chaotic system. Chaotic sequences *x* and *y* are generated by iteration.

**Step 1:** Generate the key image

A key image (*K*) is generated according to Equation (4).

(4) $$pixel=\left\lfloor y\times 256\right\rfloor$$

where $\left\lfloor •\right\rfloor$ is the integer of • down, pixel is the pixel value of the key image (*K*), with its value ranging from 0 to 255 as an integer. A key image (*K*) of resolution M × N is obtained.

**Step 2:** DNA encoding

After the pixel values of the key image (*K*) and the plain image (*P*) have been converted from decimal to binary, each line is encoded in accordance with a distinct set of rules, thereby obtaining the corresponding encoded key image ($K_{DNA}$) and encoded plain image ($P_{DNA}$). The size is M × N × 4. The specific coding rule that governs this process is determined by Equation (5).

(5) $$Rule=\left\lfloor x\times 8\right\rfloor +1$$

where *x* is the iteration value of the logistic chaotic map. Rule represents a DNA encoding rule and is an integer within the range of 1 to 8, as shown in Table 1.

**Step 3:** DNA operation

DNA operations (XOR, addition, and subtraction) are performed on the $P_{DNA}$ and the $K_{DNA}$, formulated as Equation (6).

(6) $$operation=\left\lfloor x\times 3\right\rfloor +1$$

**Step 4:** DNA decoding

The intermediate cipher image of dimensions M × N is obtained through the random DNA decoding of the aforementioned encoded intermediate image, in accordance with the tenets of Equation (5).

**Step 5:** Rotate the image

The intermediate cipher image of the preceding step is rotated 90° in a counterclockwise direction, thereby generating a new plain image. This procedure is implemented to facilitate the processing of columns.

**Step 6:** Repeat the above steps

Steps 1 to 5 steps are repeated in order to obtain the cipher image, designated as *C*.

### 2.5. Security Vulnerabilities of NEIEA-BCD

1. The mathematical expression associated with the hash of the plain image and the initial key reduces the key space to only 254 possible values, which is the key to cracking the algorithm.
2. The DNA operation is too simple, and the two DNA operations can be simplified into one.
3. The algorithm is essentially diffusion-only encryption, which does not change the position of plain pixels and does not meet the confusion condition of secure encryption.

## 3. Cryptanalysis

The origin algorithm claims to possess the ability to resist known-plaintext attacks (KPAs), as well as CPAs. Nevertheless, the results of the cryptanalysis indicate that the algorithm is not entirely secure. In light of the aforementioned three vulnerabilities, it is evident that the algorithm is unable to withstand a CPA. To this end, four distinct images are employed in order to launch a CPA, thereby enabling the subsequent recovery of the original plain image.

### 3.1. CPA

In this section, the security of the algorithm is subjected to further analysis, and potential vulnerabilities are identified. In accordance with Kerckhoffs’ principle, the security of an encryption scheme should not be contingent upon the confidentiality of the scheme itself but, rather, upon the secrecy of the key. A CPA is a cryptanalytic model that assumes that an attacker has the ability to temporarily access the encryption machine, select any plaintext that is beneficial to the attack, and subsequently obtain the corresponding ciphertext.

### 3.2. Four Properties of Our Cryptanalysis

Below, we summarize several relevant properties based on this image encryption algorithm that are relevant to our attacking of this algorithm.

**Property** **1.** *$x_{0}$ can be cracked by a brute-force attack.*

**Proof.**  An examination of Equation (3) reveals that the maximum 8-bit binary number that can be obtained after specific operations on $d_{1}$, $d_{2}$, $d_{3}$, and $d_{4}$ is 255. Consequently, it is not meaningful to take 256 modulo. Therefore, Equation (3) can be regarded as an equivalent representation of Equation (7).

(7) $$x_{0}=\left(d_{1}⊕d_{2}⊕d_{3}⊕d_{4}\right)/255$$

From Equation (7), we can see that the result after the XOR of $d_{1}$, $d_{2}$, $d_{3}$, and $d_{4}$ ranges from 0 to 255, where 0 and 255 are discarded because the initial value of the chaotic sequence cannot be 0 or 1. Therefor, there are 254 possible results from 1 to 254, i.e., $x_{0}\in \{\frac{1}{255},\frac{2}{255},$…$,\frac{253}{255},\frac{254}{255}\}$, so $x_{0}$ is insecure and can be cracked by a brute-force attack.    □

**Property** **2.** *Given the same $x_{0}$, the chaotic sequence of x and y is exactly the same.*

**Proof.**  If a special plain image is constructed such that the $x_{0}$ obtained from this image is equal to the $x_{0}$ obtained from the original plain image, the algorithm can be regarded as a plaintext-independent cryptographic algorithm. Given the same initial key condition, *x* and *y* are the same.    □

**Property** **3.** *There is a fixed correspondence between the DNA sequences of $C_{DNA}$ and $P_{DNA}$ at the same position.*

**Proof.**  This algorithm is essentially a diffusion-only algorithm. It only changes the size of the pixel value without changing its position. The counterclockwise image rotation in the sixth step is intended to encrypt the column, but although the position of the pixel is changed, it has no actual encryption effect, and the process can be resumed by reversing the rotation during cryptanalysis. In fact, the encryption algorithm can be thought of as the process of encrypting one DNA sequence into another. The encryption process can be expressed as follows:

$$P_{DNA}⊗K_{1DNA}⊗K_{2DNA}=C_{DNA}$$

where ⊗ represents the DNA operation process and $C_{DNA}$ is the DNA encoded in the ciphertext image. The encryption algorithm performs two DNA operations, but it obeys the exchange law, which can be simplified to one DNA operation:

$$P_{DNA}⊗{K^{\prime }}_{DNA}=C_{DNA}$$

We construct four special plain images ($P^{0}$, $P^{85}$, $P^{170}$, and $P^{255}$, with pixel values of 0, 85, 170, and 255, respectively) because these images all contain data encoded as ‘A’, ‘G’, ‘C’, and ‘T’ necessary to obtain the corresponding ciphertext images ($C^{0}$, $C^{85}$, $C^{170}$, and $C^{255}$).

**Figure 2:**
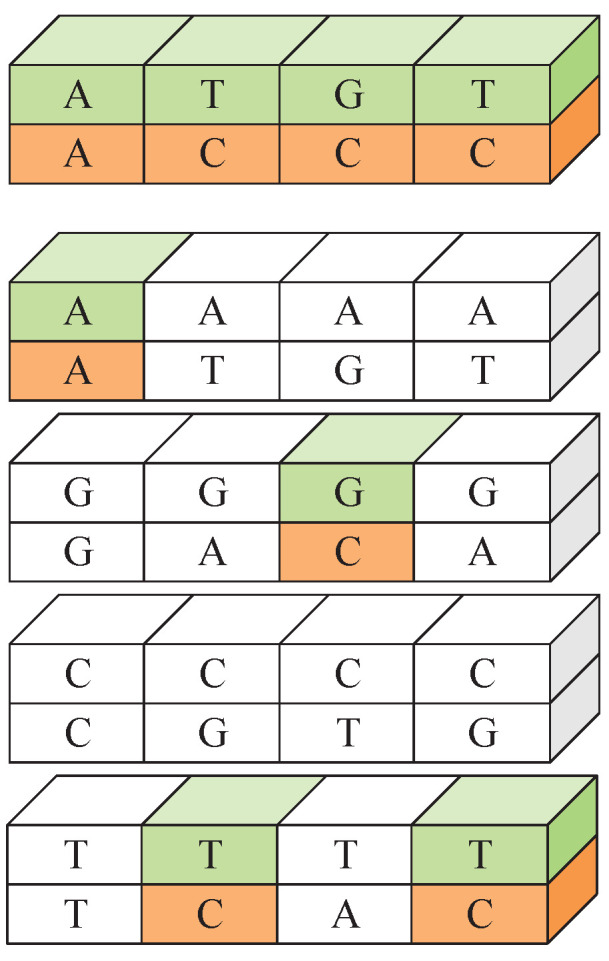
DNA sequence recovery.

By comparing the DNA sequence of *C* with that of $C^{0}$, $C^{85}$, $C^{170}$, and $C^{255}$ at the same position, the DNA sequence of the plain image at that point can be restored. Similarly, we can restore DNA sequences at other positions in this way. The recovery process is illustrated in Figure 2.    □

**Property** **4.** *When the same initial key is used to modify the pixel value at a given point in the plaintext image, the resulting ciphertext pixel value at that point is affected, while the pixel values at other locations in the image remain unaltered.*

**Proof.**  There may be different initial keys ($x_{0}$) for different images. Therefore, we need to find a special plain image ($P_{0}^{\prime }$) with the same initial key ($x_{0}$) as the encrypted image ($P_{0}$) to implement the chosen plaintext attack. We control the size of the initial key ($x_{0}$) by changing the pixel value of a point in $P_{0}^{\prime }$ to make it have the same $x_{0}$ as $P_{0}$. We prove that a change in pixel value at a certain position not spreading to other positions is also one of the necessary conditions for constructing $P_{0}^{\prime }$. Taking the above all-0 image as an example, the pixel value of $P_{0}$ on (1,1) is changed to 1 to obtain $P_{0}^{\prime }$ and the corresponding cipher image $C_{0}^{\prime }$. Suppose that $P_{0}$ and $P_{0}^{\prime }$ have the same initial key after the MD5 operation. It can be found that the pixel values of $C_{0}^{\prime }$ and $C_{0}$ are not equal at position (3,1). In other words, modifying the value of a single plain pixel has a negligible effect on cryptanalytic breaking.

$${P_{0}}^{\prime }=\left[\begin{array}{ccc} 1 & 0 & 0\\ 0 & 0 & 0\\ 0 & 0 & 0 \end{array}\right]\to {C_{0}}^{\prime }=\left[\begin{array}{ccc} 55 & 127 & 163\\ 29 & 5 & 162\\ 7 & 144 & 176 \end{array}\right]$$

   □

### 3.3. Three Steps of Cryptanalysis

In accordance with the aforementioned four properties, we employ a CPA to breach the encryption algorithm, subsequently delineating the requisite cracking steps.

**Step 1.** Construct the special plain image

How to construct a suitable special plain image is the most important step in the cryptanalysis process, which directly affects whether we can successfully crack the image encryption algorithm. In most image encryption algorithms, the initial key is fixed, but using the key associated with the plaintext, the initial key can be considered to be constantly changing. Dynamic keys can effectively resist a the chosen plaintext attack. However, the number of dynamic keys is not infinite. We can reconstruct the key space by constructing a key library that records all possible values of dynamic keys. In the NIEEC-BCD algorithm, the number of dynamic keys is only 254, and the number of keys associated with plaintext is very small, so we can construct this keys library. We construct four special plain images, and the $x_{0}$ obtained by them is required to be equal to that of the plain image (C). However, we do not know the initial key ($x_{0}$) of plain image C in advance, so we need to traverse all possible values of $x_{0}$ to obtain the desired $x_{0}$. Finally, 4 × 254 plain images are constructed, and the construction process is as shown in Algorithm 1.
**Algorithm 1** Construct special plain images**Input:** $P_{0}$**Output:** $P_{0}^{\left(I\right)},I=1,2,\cdots ,254$  1:$L=\left\{l_{1},l_{2},\cdots ,l_{254}\right\}=\left\{1,2,\cdots ,254\right\}$  2:$S=\left\{s_{1},s_{2},\cdots ,s_{254}\right\}=\left\{0,0,\cdots 0\right\}$  3:**if** S≠L **then**  4:     **for** i← to MN **do**  5:         $P_{0}^{\left(I\right)}\leftarrow P_{0}$  6:         $P_{0}^{\left(I\right)}\left(i\right)\leftarrow 1$  7:         $Value\leftarrow Hash(P_{0}^{\left(I\right)})$  8:         $x_{0}\leftarrow attack\left(Value\right)\times 255$  9:         $s_{x_{0}}\leftarrow x_{0}$  10:        $I\leftarrow x_{0}$  11:     **end for**  12:**end if**        **return** $P_{0}^{\left(I\right)}$

Where *i* is the image index value, Hash(⊙) is the hash function for ⊙, and attack(⊙) is the calculation for ⊙ using Equation (7).

**Step 2.** Get the special ciphertext image

The CPA is employed to input the special plain image constructed in the preceding step into the encryption apparatus, thereby generating the corresponding ciphertext images, designated as $C_{0}$, $C_{85}$, $C_{170}$, and $C_{255}$. These ciphertexts are subsequently utilized to recover the plain image in the subsequent step.

**Step 3.** Recover the plain image

According to Property 3, in the DNA-domain encryption process, there is a fixed binary value substitution relationship between the 2-bit input and output at any location. First, *C*, $C_{0}$, $C_{85}$, $C_{170}$, and $C_{255}$ are encoded by DNA according to rule 2 to obtain $C_{DNA}$, $C_{DNA}^{0}$, $C_{DNA}^{85}$, $C_{DNA}^{170}$, and $C_{DNA}^{255}$. Then, we get $P_{DNA}^{\prime }$ according to Algorithm 2.
**Algorithm 2** Get the $P_{DNA}^{\prime }$**Input:** $C_{DNA}$, $C_{DNA}^{0}$, $C_{DNA}^{85}$, $C_{DNA}^{170}$ and $C_{DNA}^{255}$**Output:** $P_{DNA}^{\prime }$  1:**for** *i*←1 to 4MN **do**  2:     **if** $C_{DNA}\left(i\right)=C_{2-bit}^{0}\left(i\right)$ **then**  3:          $P_{DNA}^{\prime }\left(i\right)$←A  4:     **else if** $C_{DNA}\left(i\right)=C_{2-bit}^{85}\left(i\right)$ **then**  5:          $P_{DNA}^{\prime }\left(i\right)$←G  6:     **else if** $C_{DNA}\left(i\right)=C_{2-bit}^{170}\left(i\right)$ **then**  7:          $P_{DNA}^{\prime }\left(i\right)$←C  8:     **else if** $C_{DNA}\left(i\right)=C_{2-bit}^{255}\left(i\right)$ **then**  9:          $P_{DNA}^{\prime }\left(i\right)$←T  10:     **end if**  11:**end for**       **return** $P_{DNA}^{\prime }$

After obtaining $P_{DNA}^{\prime }$, *P* is obtained by DNA decoding $P_{DNA}^{\prime }$ according to rule2, flipping *P* by 90 degrees so that the original image is successfully restored.

### 3.4. Experimental Setup

To verify the feasibility of the cryptanalysis scheme, we strictly follow the steps of the original encryption algorithm reported in [21] and use Matlab R2022b to perform experimental simulation verification of the cryptanalysis system. To ensure the integrity of the cryptanalysis work, we use the initial key that is consistent with the original paper, where μ=3.99999999 and p=0.25678900. We performed cryptanalysis experiments on some classical images.

**Figure 3:**
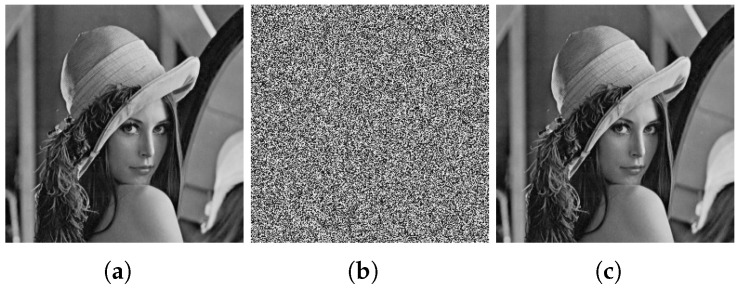

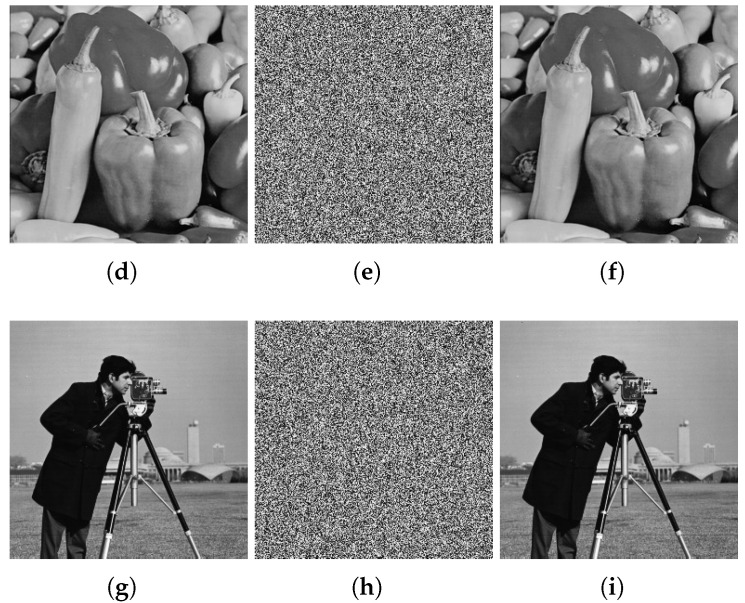
Cryptanalysis result. (**a**) Plain image of Lenna. (**b**) Cipher image of Lenna. (**c**) Recovered image of Lenna. (**d**) Plain image of peppers. (**e**) Cipher image of peppers. (**f**) Recovered image of peppers. (**g**) Plain image of camera. (**h**) Cipher image of camera. (**i**) Recovered image of camera.

The result of the cryptanalysis is shown in Figure 3, and in the following, we take the Lenna graph, a 256 × 256 grayscale image, as an example to experimentally verify the above attack process.

**Step 1.** Construct the special plain images

**Figure 4:**
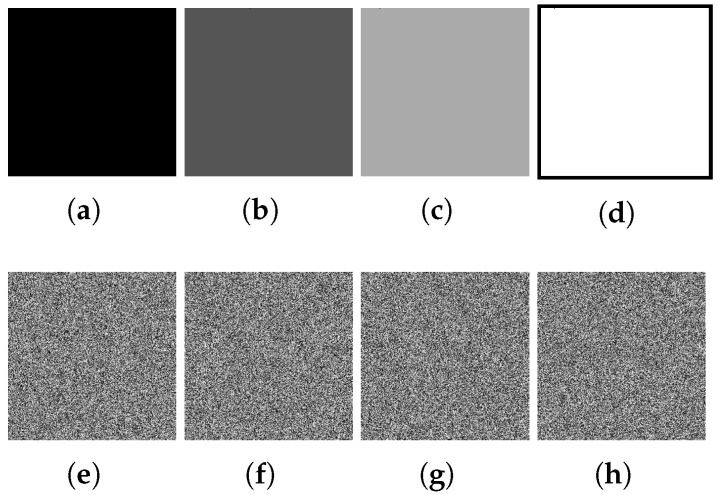
Constructed special plain image and its encrypted image.(**a**) $P_{0}$. (**b**) $P_{85}$. (**c**) $P_{170}$. (**d**) $P_{255}$. (**e**) $C_{0}$. (**f**) $C_{85}$. (**g**) $C_{170}$. (**h**) $C_{255}$.

We construct four special plain images $(P_{0}$, $P_{85}$, $P_{170}$, and $P_{255}$, where $P_{0}$ is the all-0 image (divided by (1, 243) is 1), $P_{85}$ is the all-85 image (divided by (1, 100) is 1), $P_{170}$ is the all-170 image (divided by (1, 29) is 1), and $P_{255}$ is the all-255 image (divided by (1, 21) is 1)). The hash value of $P_{0}$ is d16104f89668edb2e55a7995e8c27973, that of $P_{85}$ is 9c415fce44f551a71a83a4d809af116f, that of $P_{170}$ is 858de3a7fe0a9fd522b896124fbea7d6, and that of $P_{255}$ is 6e87d075002e35757099d0a1a2806faf. After the calculation of Equation (3), $x_{0}$ is 0.298039215686 2745, and all hash values calculated by Equation (3) are equal to the $x_{0}$ obtained by the hash value of the Lenna image calculated by Equation (3), which is 0.2980392156862745. The constructed plain image is shown in Figure 4.

**Step 2.** Encrypt the special plain image

The plain image constructed in step 1 is put into the encryption machine to obtain the corresponding ciphertexts ($C_{0}$, $C_{85}$, $C_{170}$, and $C_{255}$), which are used for subsequent attacks. The ciphertext is shown in Figure 4.

**Step 3.** Recover the plain image

The ciphertext image and the attack ciphertext image obtained in step 2 are compared and mapped using Algorithm 2 to recover the plain image. A comparison of the restored plain image with the original plain image reveals that only three pixels differ—specifically, (1, 21), (1, 29), and (1, 243). The recovered image is illustrated in Figure 3.

**Figure 5:**
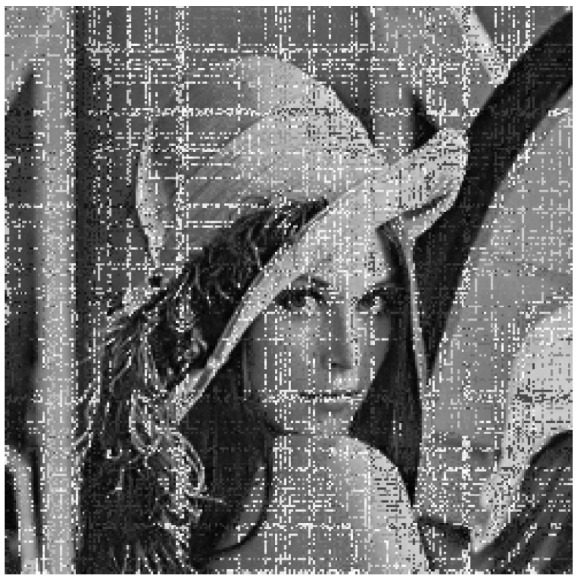
Recovered Lenna image with $P_{0}$.

In addition, only one $P_{0}$ image is used to restore the original image. Due to the asymmetry of the subtraction in the DNA operation, the recovery of the DNA sequence after the subtraction operation fails. Finally, 83.79 percent of the plain image is successfully recovered using only one image, and the recovery effect is shown in Figure 5.

## 4. Improvement of the Original Algorithm

Through the above analysis, we find that NEIEA-BCD has security vulnerabilities and cannot resist CPAs. In this chapter, we propose an improved logistic chaotic map with random bit-position perturbation named BPP-Logistic Chaotic, which has a wider parameter range, stronger robustness, and a larger Lyapunov exponent. Finally, we improve the original algorithm.

### 4.1. BPP-Logistic Chaotic

**Figure 6:**
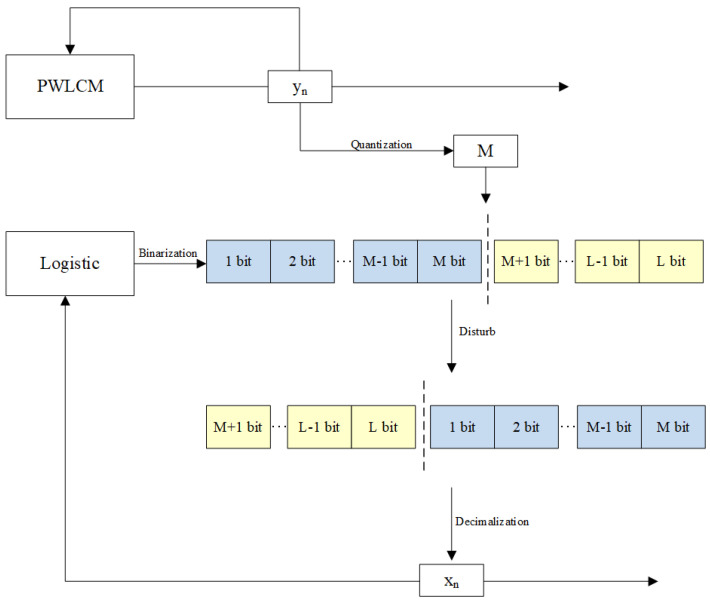
BPP-Logistic Chaotic.

Inspired by image permutation, we propose an improved logistic chaotic system by randomly perturbing the state variables of the chaotic system, the schematic diagram of which is shown in Figure 6.

First, the initial value is injected into the PWLCM and logistic chaotic system for iteration. The chaotic sequence of the PWLCM iteration is quantized to obtain a decimal integer (M(M≤L)), and the chaotic sequence of the logistic chaotic system iteration is binarized to obtain a binary value of *L* bits. Then, the positions of the first bit (*M*) and the last bit (L−M) of the binary value are interchanged to realize the perturbation, and the obtained base number is converted into decimal output.

#### 4.1.1. Analysis of Chaotic Characteristics

**Figure 7:**
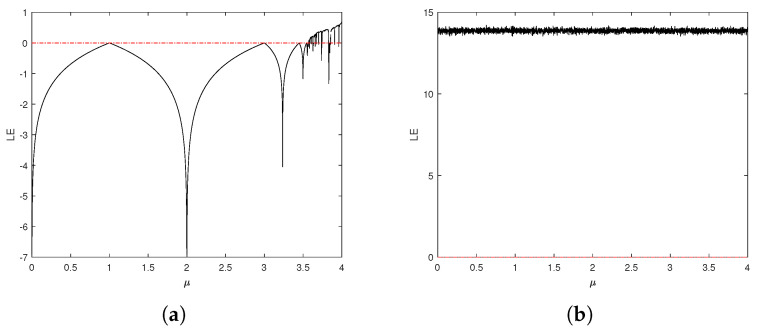
Lyapunov exponent. (**a**) LE of the logistic system. (**b**) LE of BPP-Logistic.

For a discrete chaotic system, the Lyapunov exponent is the key index to determine whether a system has chaotic behavior. It represents the numerical characteristics of the average exponential divergence rate of adjacent trajectories in phase space. If the Lyapunov exponent is greater than 0, the system is considered to be chaotic. The larger the positive Lyapunov exponent, the more significant the chaotic behavior of the system. The LE of the improved logistic system is shown in Figure 7. It can be seen that when μ∈(0,4], the LE of this system remains around 14. In comparison to the original logistic system, the parameters of the enhanced system in a chaotic state exhibit a broader range, accompanied by a higher LE value.

**Figure 8:**
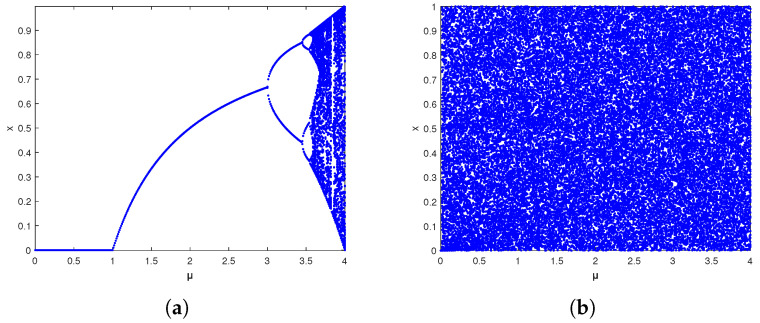
Bifurcation diagram. (**a**) Bifurcation diagram of the logistic system. (**b**) Bifurcation diagram of BPP-Logistic.

The bifurcation diagram can intuitively judge the stability of the system under different parameters. The bifurcation diagram in the parameter range of μ∈(0,4] is shown in Figure 8. The original logistic system has chaotic properties only when μ∈(3.5699456,4], while the improved logistic chaotic system is chaotic in the parameter range of μ∈(0,4], so it is robust and has a wider range of parameters.

#### 4.1.2. Frequency Histogram and Trajectory

**Figure 9:**
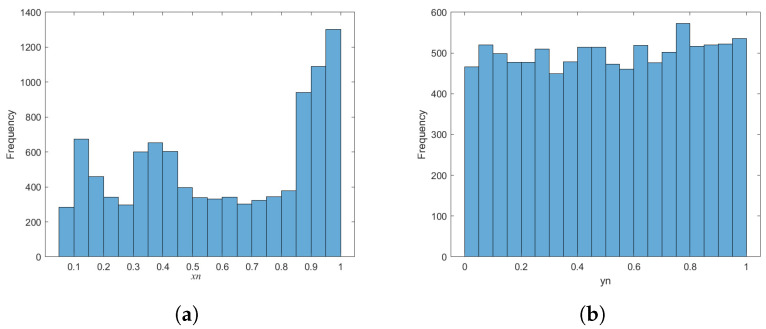
Frequency histogram. (**a**) Frequency histogram of the logistic system. (**b**) Frequency histogram of BPP-Logistic.

The frequency histogram can be used to show the frequency of each value of the chaotic sequence and to intuitively evaluate the statistical characteristics of the chaotic system. A good chaotic system should have a uniform frequency histogram. In the case of the same initial parameter (μ=3.9) and the same initial value ($x_{0}=0.12345$), the original logistic system and the improved BPP-Logistic are iterated 10,000 times to obtain the chaotic sequences ($x_{n}$ and $y_{n}$). Then, the frequency statistics of xn and yn are calculated. The resulting frequency histogram is shown in Figure 9. It can be found from the figure that the frequency of the chaotic sequence ($x_{n}$) of the original logistic chaotic system in the interval of [0.9–1] is close to 2400 times, which is much higher than the frequency of other intervals, so it may be attacked by statistical analysis in cryptography. The improved BPP-Logistic chaotic sequence ($y_{n}$) has a more uniform distribution in the range of [0, 1], and has better numerical statistical characteristics.

**Figure 10:**
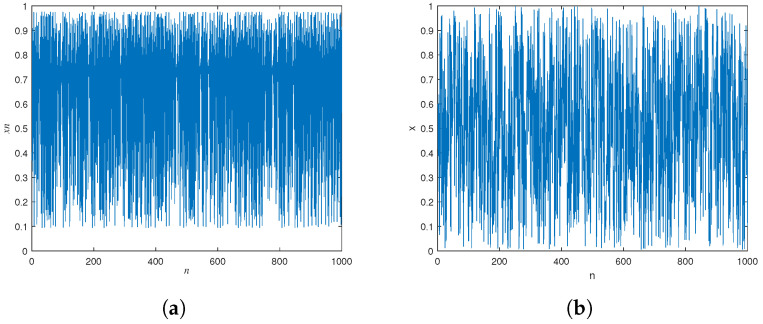
Trajectory. (**a**) Trajectory of the logistic system. (**b**) Trajectory of BPP-Logistic.

The trajectory diagram shows the complex and random behavior of the system in phase space over time. Figure 10 is the trajectory diagram obtained by 1000 iterations with a parameter of μ=3.9. It can be seen that the dynamic behavior of the improved logistic system is more complex in the 0–1 range.

### 4.2. Improved NEIEA-BCD

In this section, we improve the original algorithm. In addition to replacing the logistic chaotic system in NEIEA-BCD with BPP-Logistic, we also improve it from two perspectives. First, we improve the key generation mechanism to make it have a larger key space. Secondly, a new permutation operation is proposed.

#### 4.2.1. Improvement of the Initial Key

In the original algorithm, the first 32 bits of the hash value of the plain image are used, and there are only 8 bits after the XOR operation in Equation (3). The key space associated with the plaintext is only $2^{8}$, so it can be broken by brute force. To this end, we improve the process of initial key generation. The initial key consists of the following parts: initial values ($x_{0}$ and $y_{0}$) of the improved logistic chaotic map and control parameters (μ and *p*), where $x_{0}$ and $y_{0}$ are calculated as shown in Equation (8).

(8) $$\begin{array}{c} x_{0}=P_{hash1}\times 2^{-64}\\ y_{0}=P_{hash2}\times 2^{-64} \end{array}$$

where $P_{hash1}$ is the first 64 bits of the hash value of the plain image; $P_{hash2}$ is the last 64 bits of the hash value of the plain image; its key space is $2^{64}\times 2^{64}\times 3\times 10^{16}\times 10^{16}$, which is greater than $2^{100}$; and the key space associated with the plaintext is $2^{128}$. Therefore, the key space is secure enough.

#### 4.2.2. Improvement of the Permutation Process

**Figure 11:**
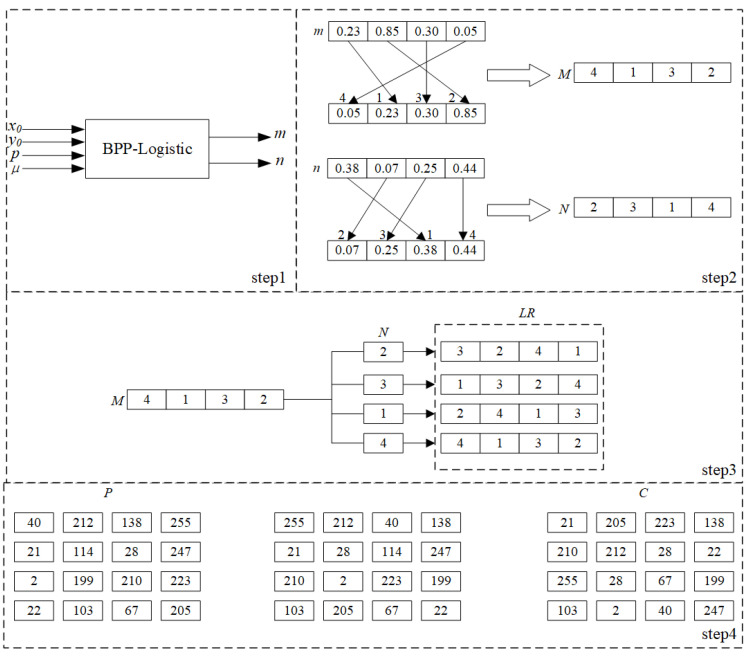
Latin matrix permutation process.

There is no permutation process in the original algorithm. Therefore, its security is questioned, and it cannot resist a cropping attack. Therefore, we add a permutation process before the DNA encoding step to improve the security of the original algorithm. Here, we use the chaotic sequence to construct the Latin matrix for permutation. Since each different element of the Latin matrix can only appear once in the same row or column, a very good permutation effect can be obtained. Taking an image with a resolution of 4 × 4 as an example, the permutation process is shown in the Figure 11.

First, the BPP-Logistic system is used to generate two sequences of length 4:*m*,*n*. Then, the two sequences of *m* and *n* are sorted in ascending order, and the corresponding index vector (*M*, *N*) is obtained. In the third step, M is cyclically shifted to the right four times according to the elements of N, and four rows of elements are obtained. The Latin square matrix (LR) is obtained by combining the elements of these four rows. Finally, the elements of each row of plain image P are reordered according to LR, then sorted by column to obtain the cipher image (C).

#### 4.2.3. Other Improvements

**Figure 12:**
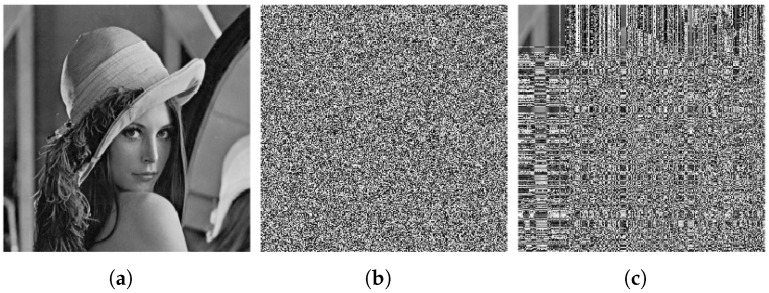
Sensibility of secret keys. (**a**) Plain image of Lenna. (**b**) Cipher image of Lenna. (**c**) Decrypted image with $x_{0}^{\prime }$.

In the analysis of the original algorithm, we find that some of the previous values are abandoned to avoid transient effects when using chaos, which make it not sensitive to the key. This leads to the disclosure of some private information when using a key with a small difference from the initial key for decryption. Figure 12 shows the image using decrypted using the key with $x_{0}^{\prime }=x_{0}+10^{-15}$. To avoid this, we discard the first 100 iterations to avoid transient effects.

In addition to this, we improve the pairs of Equations (4)–(6) to obtain better encryption results. The improved formula is as follows:

$$\begin{array}{c} pixel=floor(\left(x\times 10^{10}-floor\left(x\times 10^{10}\right)\right)\times 256)\\ Rule=floor(\left(x\times 10^{10}-floor\left(x\times 10^{10}\right)\right)\times 8)+1\\ operation=floor(\left(x\times 10^{10}-floor\left(x\times 10^{10}\right)\right)\times 3)+1 \end{array}$$

where floor means rounded down.

The NIST (National Institute of Standards and Technology) test suite is a suite of statistical testing tools for evaluating the quality of the output of a random number generator (RNG). These tests are designed to detect whether a sequence of random numbers has the expected randomness and unpredictability. We conduct the NIST randomness test on the above pixel using 1000 groups of 100,000 bits of data using the NIST800-22 statistical test suite. When the calculated *p* value is greater than 0.01, the randomness test is complete. The test results are shown in the Table 3.

**Figure 13:**
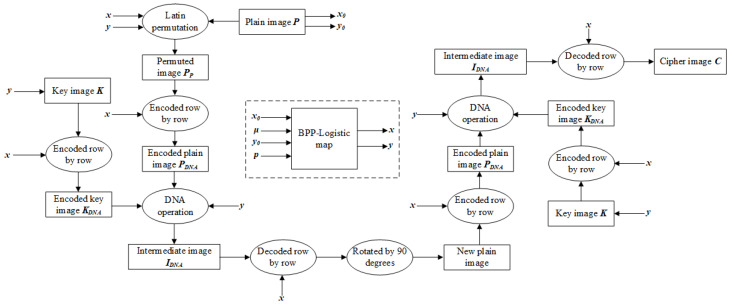
Improved NEIEA-BCD.

In this section, the chaotic system and encryption algorithm used by NEIEA-BCD are improved to increase security in applications. The improved flow chart of NEIEA-BCD is shown in Figure 13.

### 4.3. Security Analysis of Improved NEIEA-BCD

#### 4.3.1. Statistical Analysis

**Figure 14:**
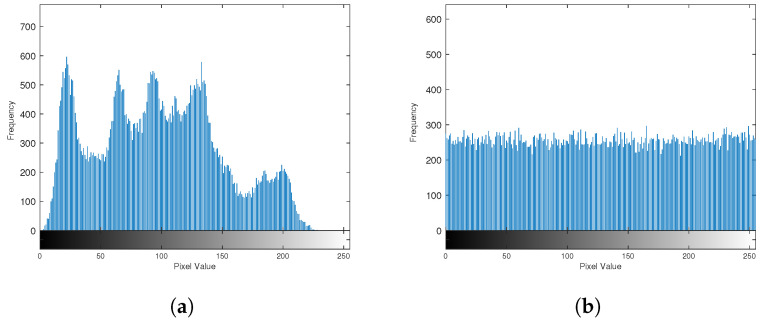
Histogram. (**a**) Histogram of Lenna. (**b**) Histogram of cipher image.

Statistical analysis is a necessary condition for the security of image encryption algorithms. Histogram and correlation analyses are important indicators to measure statistical characteristics. A more uniform histogram of the encrypted image indicates a more effective encryption process. Figure 14 shows the statistical histogram of the encryption of the Lenna image.

**Figure 15:**
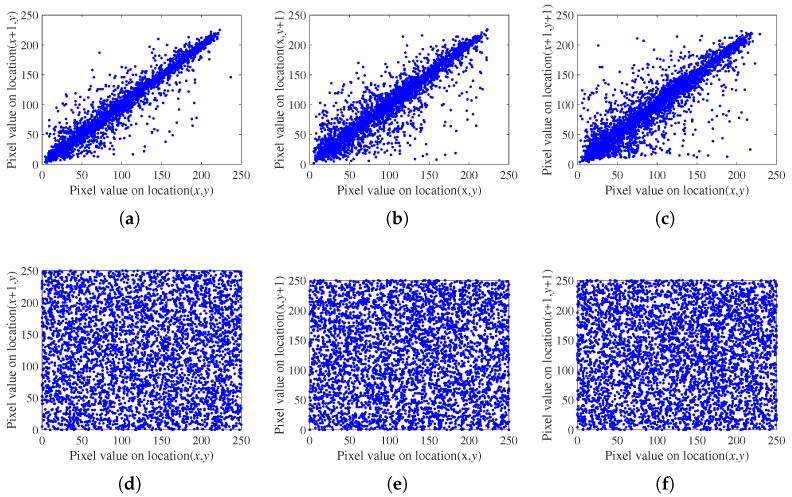
Correlations of adjacent pixels. (**a**) Horizontal direction of the plain image. (**b**) Vertical direction of the plain image. (**c**) Diagonal direction of the plain image. (**d**) Horizontal direction of the cipher image. (**e**) Vertical direction of the cipher image. (**f**) Diagonal direction of the cipher image.

The image exhibits a significant degree of correlation among neighboring pixels; therefore, an effective encryption algorithm should disrupt this correlation. Figure 15 shows the correlation analysis of 5000 pairs of randomly selected adjacent pixels, examining their relationships in the horizontal, vertical, and diagonal orientations.

The correlation coefficient is used as a quantitative parameter to study the correlation, with its calculation formula given below.

$$r_{x,y}=\frac{\mathrm{cov}(x,y)}{\sqrt{D(x)}\sqrt{D(x)}}$$

The average values of the correlation coefficients obtained by repeating the experiment 100 times are shown in Table 4.

#### 4.3.2. Analysis of Key Sensitivity

**Figure 16:**
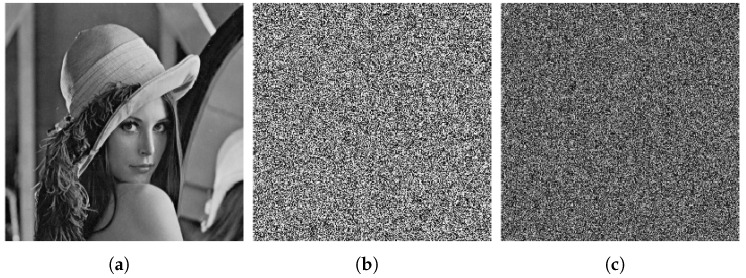

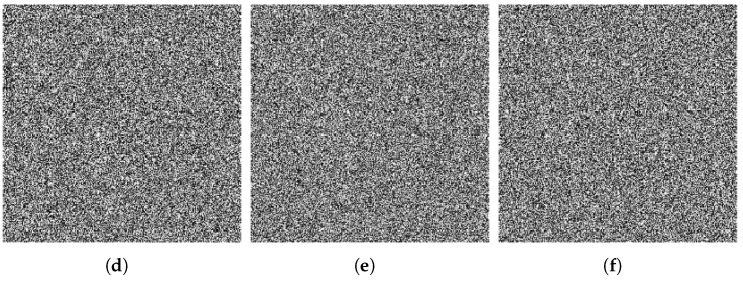
Key sensitivity. (**a**) Plain image. (**b**) Cipher image. (**c**) Image decrypted with $x_{1}$. (**d**) Image decrypted with $y_{1}$. (**e**) Image decrypted with μ. (**f**) Image decrypted with *p*.

The improved algorithm abandons the first 100 iterations of the chaotic system to avoid the influence of transient effects on encryption, so it is more sensitive to the initial key. Figure 16 show the images decrypted using the incorrect keys ($x_{1}=x_{0}+10^{-15}$, $y_{1}=y_{0}+10^{-15}$, $\mu_{1}=\mu +10^{-15}$, and $p_{1}=p+10^{-15}$).

#### 4.3.3. Information Entropy

Information entropy serves as a significant metric for assessing the complexity of an image, and the equation for its calculation is presented below.

$$H\left(I\right)=-\sum_{i=0}^{L-1}p\left(i\right)\log_{2}p\left(i\right)$$

where H(I) represents the information entropy, p(i) represents the probability that the image pixel value is the occurrence of *i*, and *L* represents the total number of gray levels. in this paper, *L* = 256. The closer *H* is to 8, the higher its complexity is. From Table 5, it can be found that the information entropy after encryption is very close to 8.

#### 4.3.4. Analysis of Resistance to Cropping Attacks

**Figure 17:**
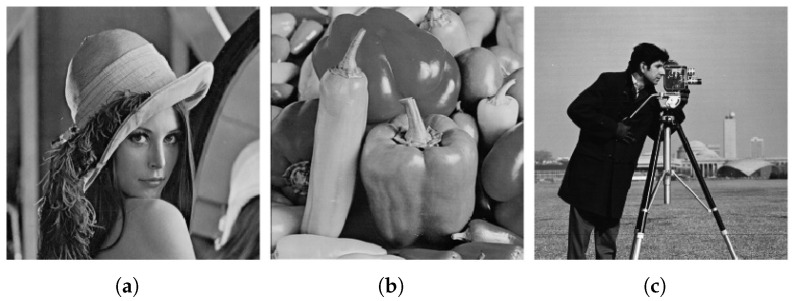

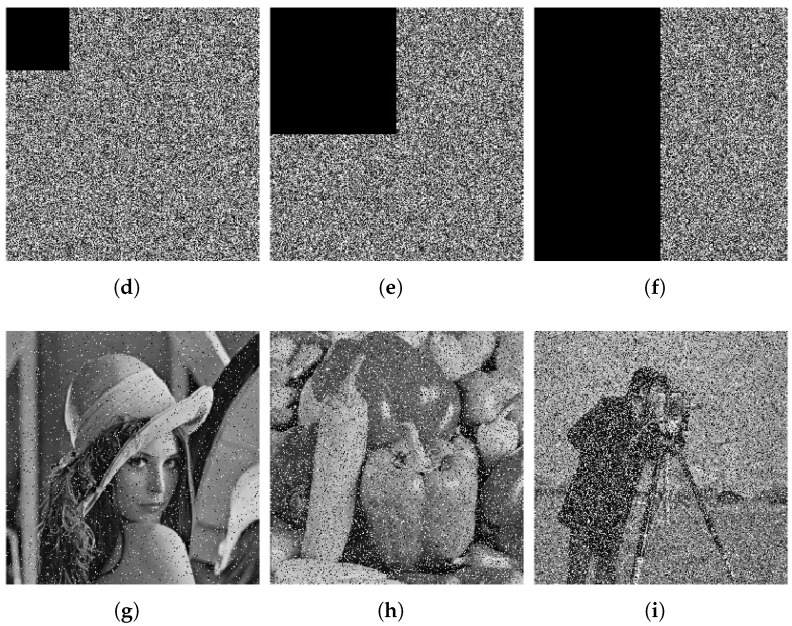
Resistance to cropping attacks. (**a**) Plain image of Lenna. (**b**) Plain image of peppers. (**c**) Plain image of camera. (**d**) Cropping area of 64 × 64. (**e**) Cropping area of 128 × 128. (**f**) Cropping area of 128 × 256. (**g**) Decrypted image of Lenna. (**h**) Decrypted image of peppers. (**i**) Decrypted image of camera.

Cropping attacks are a common form of cryptographic attack that introduce noise into specific regions of an image, thereby compromising the integrity of the ciphertext and rendering it unintelligible to the intended recipient. The ability of an encryption algorithm to resist such attacks is a crucial indicator of its quality. The original algorithm is susceptible to cropping attacks, whereas the enhanced algorithm demonstrates enhanced resilience to these attacks. Figure 17 shows the excellent anti-cropping ability of the improved algorithm.

#### 4.3.5. Analysis of Resistance to Differential Attack

The Number of Pixels Change Rate (NPCR) and Unified Average Changing Intensity (UACI) are essential metrics for evaluating the resilience of encryption algorithms against differential attacks. The NPCR indicates the proportion of the total number of pixels in two encrypted images where the pixel values are not equal at the same position. By merely altering a single pixel value in the plain image, we compare the pixel change rate of the two encrypted images. The specific calculation formula is presented as follows, and its ideal value is 99.6094.

$$D\left(i,j\right)=\left\{\begin{array}{c} D\left(i,j\right)=0,C_{1}\left(i,j\right)=C_{2}\left(i,j\right)\\ D\left(i,j\right)=1,C_{1}\left(i,j\right)\neq C_{2}\left(i,j\right) \end{array}\right.$$

$$NPCR=\frac{\sum_{i=0}^{M-1}\sum_{j=0}^{N-1}D(i,j)}{M\times N}\times 100\%$$

Specifically, C(i,j) represents the pixel value of the encrypted image at (*i*, *j*). UACI quantifies the extent of variation in the encrypted pixel values across different plain images, with its calculation equation outlined below. The ideal value is 33.4635%.

$$UACI=\frac{1}{M\times N}\left(\sum_{i,j}\frac{\left|C_{1}\left(i,j\right)-C_{2}\left(i,j\right)\right|}{255}\right)\times 100\%$$

In Table 6, the NPCR and UACI of the image averaged over multiple experiments are shown to be close to the ideal value, indicating good resistance to differential attacks.

#### 4.3.6. Resisting Cryptanalysis

In the original algorithm, although the hash value of the plain image is associated with the key, its correlation degree remains low, rendering it insufficient to withstand a CPA. The key ($x_{0}$) can be easily compromised by brute-force methods, and once the encryption algorithm dependent on $x_{0}$ is breached, it devolves into a key-independent encryption scheme that is vulnerable to CPAs. In contrast, the improved encryption algorithm leverages a stronger correlation between plaintext and the key to effectively counteract CPAs. This approach may inspire new strategies for cryptanalysis practitioners, while designers should take care to avoid such pitfalls.

#### 4.3.7. Analysis of Computational Complexity and Runtime

Computational complexity analysis and runtime analysis are important indicators for evaluating the efficiency of an algorithm. In the NEIEA-BCD encryption algorithm, there are four parts involving complexity calculation: the complexity of generating the key image is O(M × N), the complexity of DNA encoding is O(4 × M × N), the complexity of the DNA operation is O(4 × M × N), and the complexity of DNA decoding is O(M × N). Since these four processes are executed in sequence, the computational complexity of the NEIEA-BCD encryption algorithm is O(4 × M × N). The improved NEIEA-BCD encryption algorithm adds a permutation operation, and the complexity of the permutation operation is O(M × N), so the computational complexity of the improved encryption algorithm is not increased, and it is still O(4 × M × N). Table 7 shows a comparison of the running times for different images. Through the comparison, it can be found that the improved encryption algorithm only sacrifices a small part of its efficiency but with greatly improved security of the encryption system.

## 5. Conclusions

In this paper, a security analysis of NEIEA-BCD is carried out. From the perspective of cryptanalysis, the results demonstrate that the original algorithm is susceptible to security vulnerabilities. While the encryption algorithm is capable of resisting CPAs by associating the key with the plaintext, the mathematical operation of association is too simple, resulting in the key being limited to between 1/255 and 254/255. Consequently, it can be broken by a brute-forced attack. Furthermore, the algorithm is, in fact, a diffusion-only encryption algorithm. Despite the increased complexity of the algorithm resulting from the use of DNA coding, an equivalent diffusion key exists. In light of the above considerations, this paper presents a methodology based on CPA to crack the original algorithm. Due to the mechanism by which the key is associated with the plaintext, there are 254 possibilities for the key. We construct 254 × 4 grayscale images of size M × N, and the complexity of the attack is O(4 × 254). The cryptanalysis presented in this paper offers valuable insights for designers of chaotic image encryption algorithms, facilitating the development of more secure algorithms. In addition, we improve the original algorithm to resist this attack.

## Figures and Tables

**Table 1:** The coding rules of DNA.

Rule (R)	R1	R2	R3	R4	R5	R6	R7	R8
00	A	A	T	T	C	C	G	G
01	C	G	C	G	A	T	A	T
10	G	C	G	C	T	A	T	A
11	T	T	A	A	G	G	C	C

**Table 2:** Detailed DNA operations.

XOR	A	C	T	G	+	A	C	T	G	−	A	C	T	G
A	A	C	T	G	A	C	A	G	T	A	C	G	A	T
T	T	G	A	C	T	G	T	C	A	T	G	T	C	A
C	C	A	G	T	C	A	C	T	G	C	A	C	T	G
G	G	T	C	A	G	T	G	A	C	G	T	A	G	C

**Table 3:** NIST test results.

Test Item	*p*-Value	Result	Pass Rate
Non-overlapping template	0.622546	Pass	0.995
Overlapping template	0.561639	Pass	0.991
FFT	0.321648	Pass	0.981
Frequency	0.292519	Pass	0.997
Approximate entropy	0.229559	Pass	0.988
Cumulative sum	0.454053	Pass	0.998
Linear complexity	0.259616	Pass	0.991
Longest run	0.352432	Pass	0.990
Random excursion	0.435787	Pass	0.994
Random excursion variant	0.406614	Pass	0.997
Block frequency	0.906069	Pass	0.994
Rank	0.904708	Pass	0.987
Runs	0.177628	Pass	0.994
Serial	0.605916	Pass	0.992
Universal	0.242986	Pass	0.982

**Table 4:** Correlation coefficients.

Image	Plain Image			Cipher Image		
	Horizontal	Vertical	Diagonal	Horizontal	Vertical	Diagonal
Lenna	0.9692	0.9404	0.9179	−0.0025	0.0023	−0.0036
Peppers	0.9698	0.9619	0.9361	0.0014	0.0066	0.0020
Camera	0.9588	0.9339	0.9078	−0.0086	−0.0007	−0.0002

**Table 5:** Information entropy.

Name	Lenna	Peppers	Camera
Plain image	7.5683	7.5797	7.0193
Cipher image	7.9971	7.9963	7.9969

**Table 6:** Information entropy.

Name	Lenna	Peppers	Camera
NPCR	99.5898	99.6074	99.5961
UACI	33.5231	33.4046	33.5208

**Table 7:** Analysis of running times.

Image	NEIEA-BCD		Improved NEIEA-BCD	
	Encryption Time (s)	Decryption Time (s)	Encryption Time (s)	Decryption Time (s)
Lenna	1.0996	1.1107	1.2726	1.2578
Peppers	1.0881	1.0674	1.2485	1.2292
Camera	1.0930	1.0908	1.2409	1.2363

## Data Availability

Data is contained within the article.
